# Accelerated Calvarial Healing in Mice Lacking Toll-Like Receptor 4

**DOI:** 10.1371/journal.pone.0046945

**Published:** 2012-10-10

**Authors:** Dan Wang, James R. Gilbert, James J. Cray, Adam A. Kubala, Melissa A. Shaw, Timothy R. Billiar, Gregory M. Cooper

**Affiliations:** 1 Department of Plastic Surgery, University of Pittsburgh, Pittsburgh, Pennsylvania, United States of America; 2 Department of Stomatology, Tenth People's Hospital of Tongji University, Shanghai, People's Republic of China; 3 Departments of Oral Biology and Orthodontics, Georgia Health Sciences University, Augusta, Georgia, United States of America; 4 Department of Surgery, University of Pittsburgh, Pittsburgh, Pennsylvania, United States of America; 5 Departments of Oral Biology and Bioengineering, University of Pittsburgh, Pittsburgh, Pennsylvania, United States of America; Charité-University Medicine Berlin, Germany

## Abstract

The bone and immune systems are closely interconnected. The immediate inflammatory response after fracture is known to trigger a healing cascade which plays an important role in bone repair. Toll-like receptor 4 (TLR4) is a member of a highly conserved receptor family and is a critical activator of the innate immune response after tissue injury. TLR4 signaling has been shown to regulate the systemic inflammatory response induced by exposed bone components during long-bone fracture. Here we tested the hypothesis that TLR4 activation affects the healing of calvarial defects. A 1.8 mm diameter calvarial defect was created in wild-type (WT) and TLR4 knockout (TLR4^−/−^) mice. Bone healing was tested using radiographic, histologic and gene expression analyses. Radiographic and histomorphometric analyses revealed that calvarial healing was accelerated in TLR4^−/−^ mice. More bone was observed in TLR4^−/−^ mice compared to WT mice at postoperative days 7 and 14, although comparable healing was achieved in both groups by day 21. Bone remodeling was detected in both groups on postoperative day 28. In TLR4^−/−^ mice compared to WT mice, gene expression analysis revealed that higher expression levels of IL-1β, IL-6, TNF-α,TGF-β1, TGF-β3, PDGF and RANKL and lower expression level of RANK were detected at earlier time points (≤ postoperative 4 days); while higher expression levels of IL-1β and lower expression levels of VEGF, RANK, RANKL and OPG were detected at late time points (> postoperative 4 days). This study provides evidence of accelerated bone healing in TLR4^−/−^ mice with earlier and higher expression of inflammatory cytokines and with increased osteoclastic activity. Further work is required to determine if this is due to inflammation driven by TLR4 activation.

## Introduction

The skeletal and immune systems are interconnected and share multiple signaling pathways [1]. The inflammatory stage of healing that occurs immediately after fracture is non-specific and shares signaling pathways with non-skeletal injuries like skin wound healing. Bone components exposed by long bone fracture possessed immunologic properties that play an important role in the induction of local, but not systemic, inflammation [2]–[3]. Inflammation promotes cell proliferation and migration into the fracture site, triggering a healing cascade within damaged bone [4]. Inflammation has a well-established role in promoting long bone regeneration. The use of nonsteroidal anti-inflammatory drugs is contra-indicated in patients with bone injuries [5]. Fracture repair is significantly delayed in COX-2^−/−^ mice, suggesting that efforts to blunt inflammation may be deleterious to fracture healing [6]. However, other studies have observed accelerated fracture repair in the absence of an adaptive immune system [7]. The role of the inflammatory response in bone regeneration is more complex than originally envisioned and is not fully understood.

Toll-like receptors (TLRs) play an essential role in innate recognition of microbial products and are critical activators of the innate immune response. More than ten TLRs have been shown to recognize distinct microbial products, such as microbial membrane lipids or nucleic acids. Another unique role of TLRs is to sense cellular stress or tissue damage by responding to endogenous ligands released from necrotic cells and damaged tissues [8]. Direct TLR signaling inhibits RANKL-mediated osteoclast differentiation, while TLR activation has also been shown to enhance osteoclast differentiation by inducing RANKL and TNF-α expression in osteoblasts [9]. Thus, the role of TLRs in bone healing remains unclear and deserves further investigation [10]–[12].

Toll-like receptor 4 (TLR4), a cell surface TLR, plays a unique role in sensing tissue damage. TLR4 signaling is activated in response to tissue injury, resulting in the induction of a sterile inflammatory cascade [13]. TLR4 has also been implicated in regulating the systemic inflammatory response following bilateral femoral fracture [14]. Whether TLR4 plays a role in the local inflammatory response to calvarial defects and the impacts of this response on healing remains unknown.

In the current study, we hypothesized that TLR4 activation affects the healing of calvarial defects, and that different gene expression patterns would be observed between wild-type (WT) and Toll-like receptor 4 mutant (TLR4^−/−^) mice during the healing process. To test this hypothesis, we assessed bone healing in small calvarial defects created in WT and TLR4^−/−^ mice using radiographic, histologic and gene expression analyses.

## Materials and Methods

### Animal care and experimental design

Wild-type C57BL-6J mice (Jackson, Bar Harbor, ME) and TLR4^−/−^ mice (from an ongoing breeding colony housed at the University of Pittsburgh) between 10 and 12 weeks of age (20–30 g) were used in this study. Mice were randomly chosen for radiographic, histological, or RT-PCR analyses. All mice were maintained in the Rangos Research Center Animal Facility, Children's Hospital of University of Pittsburgh with a 12:12 h light-dark cycle and free access to standard laboratory food and water. All procedures were carried out in accordance with the regulations regarding the care and use of experimental animals published by the National Institutes of Health and were approved by the Institutional Animal Care and Use Committee of the University of Pittsburgh.

### Calvarial defects model

Mice were anesthetized by inhalation with isoflurane (4% for induction, 2% for maintenance, Abbott Laboratories, Chicago). The scalps were depilated and cleaned prior to surgery. Under sterile conditions, the calvariae were exposed by midline scalp incision and the periosteum covering the entire parietal bone was stripped off. A circular parietal defect was made using a 1.8 mm outer diameter trephine (Fine Science Tools, Foster City, CA). The calvariae were irrigated with PBS during surgery. Following creation of the defect, the scalp was reapproximated and closed with 4-0 Vicryl resorbable sutures and 1 mg/kg ketoprofen (Fort Dodge Animal Health, Fort Dodge, IA) was administered as an analgesic immediately after surgery. All animals were euthanized by CO_2_ overdose followed by cervical dislocation at designated time points postoperatively.

### Histologic analyses

Between three and five mice from each group were euthanized on untreated day 0 and postoperative days 1, 4, 7, 14, 21 and 28. Calvariae and surrounding soft tissues (e.g., skin, brain) were harvested by cutting the skull bones anteriorly across the middle of the frontal bones and posteriorly through middle of the interparietal bone. The samples were fixed in 10% neutral buffered formalin for 24–48 h and were decalcified overnight in Cal-Ex decalcifer (Fisher Scientific, Hampton, N.H.) prior to being dehydrated through a series of alcohols and embedded in paraffin. Paraffin-embedded specimens were sectioned through the coronal plane at a thickness of 5 µm. Three regions of each defect, 50 µm apart, were cut and placed on slides (for a total of approximately 30 slides per animal). Slides were stained with Harris' hematoxylin & eosin (Surgipath Medical Industries, Richmond, IL) for conventional, qualitative bright-field light microscopy.

Histomorphometric analysis was performed to quantify the two-dimensional area of new bone formation. Healing data was calculated based on three to five slides per animal. Microscopic images of the histologic sections under 100× were analyzed using Northern Eclipse software (Empix Imagine, Inc., Mississauga, Ontario, Canada). New bone area was calculated as the sum of the areas of each bone section, including within the defect and on both sides of calvarial. Data were expressed as mean +/− SEM.

Russell-Movat pentachrome staining (American MasterTech, CA) was performed to further differentiate the following tissues within the defect: hematoma/fibrin (intense red), elastic fibers (black), cartilage (deep green), granulation/fibrous tissue (green or light blue) formation and degradation, newly-formed woven bone (yellow) and lamellar bone (red). All specimens were examined at 25×, 50×, 100× and 200× magnifications.

Immunohistochemistry stain: Sections were deparaffinized with xylenes and rehydrated through serial of EtOH dilutions to distilled H_2_O. Following the manufacturer's instructions, sections were incubated in primary goat polyclonal anti-OPN (Santa Cruz Biotechnology, Santa Cruz, CA) as a marker of osteogenic differentiation suspended at a 1∶250 dilution in 2% normal horse serum (Vector Laboratories, Burlingame, CA) for 30 min at room temperature, secondary antibody (biotinylated anti-goat, made in horse, BA-9500, Vector Laboratories, Inc. CA, USA) diluted at 1∶250 for 30 mins at room temperature, and with Streptavidin-HRP (R&D systems, Gaithersburg, MD) at dilution 1∶500 for 30 mins at room temperature. Color was developed by application of DAB kit (Vector Laboratories, Burlingame, CA). Sections were dehydrated and mounted prior to examination at 25×, 50×, 100×, and 200× magnifications.

### Radiographic analysis

Ten animals from each group were euthanized 28 days after surgery and calvariae were harvested and fixed in 10% neutral buffered formalin for 24–48 hours. Radiographs were obtained using a Faxitron MX-20 (Faxitron X-Ray, Lincolnshire, IL) with a 35 Kv exposure and a 45-second exposure time to analyze calvarial healing. The films (Eastman Kodak, Rochester, NY) were developed and scanned using a Microtek 9800 XL scanner (Microtek Lab, Inc., Fontana, CA). The scanned images were imported into Northern Eclipse software (Empix Imagine, Inc., Mississauga, Ontario, Canada). The remaining defect area was measured, and subtracted from the geometric original defect area (2.54 mm^2^) to generate the area of newly-formed bone.

### mRNA extraction and expression analysis

Five to seven mice from each group per time point were killed at day 0 before surgery and at 3 h, days 1, 2, 4, 7, 14, 21, 28 postoperatively. Samples surrounding the initial 1.8 mm defect were collected using a 5.0 mm trephine (Fine Science Tools, Foster City, CA), and included blood clots, hematoma, granulation tissue, new bone and surrounding normal bone. Samples were stored at 4°C in RNAlater solutions (Life Technologies, NY) until ready for RNA isolation. The specimens were homogenized in liquid nitrogen using a mortar and pestle, and RNA was extracted from the sample using RNAqueous-Micro Kit (Life technologies, NY) and the manufacturer's protocol. Contaminating genomic DNA was eliminated by treatment with DNAse I (Invitrogen, Life Technologies). Primers used in the study recognize IL-1α, IL-1β, IL-6, TNF-α, BMP-2, BMP-4, TGF-β1, TGF-β2, TGF-β3, VEGF, PDGF, RANK, RANKL and OPG. The housekeeping genes GAPDH and EEF2 were chosen as internal controls. RT-PCR results were analyzed by standard curve analysis. Finally, relative gene expression in WT and TLR4^−/−^ mice was compared at designated time points.

### Statistical analysis

Statistical analyses were performed using SPSS v.20.0 software (SPSS, Inc, Chicago, IL). Mean areas of newly-formed bone collected from histomophometric measurements were compared using a group by time point (2×4) two-way ANOVA followed by group by time point (1×4) split plot one-way ANOVAs to compare each group (either WT or TLR4^−/−^) over time. Post-hoc LSD tests for multiple comparisons were used to detemine significant differences among groups and time points. Independent t-tests were performed to compare histomorphometric and radiographic measurements of newly-formed bone area in WT and TLR4^−/−^ mice at each time point.

PCR data violated the assumption of homogeneity of variance, so the data was transformed using a rank transformation. Data were compared using two-way (time x group) ANOVA and are compared according to “early”(≤4 days) and “late”(>4 days) time point groups. A *p* value less than or equal to 0.05 was considered significant.

## Results

### Histologic analysis

#### Qualitative histologic analysis

**Figure 1:**
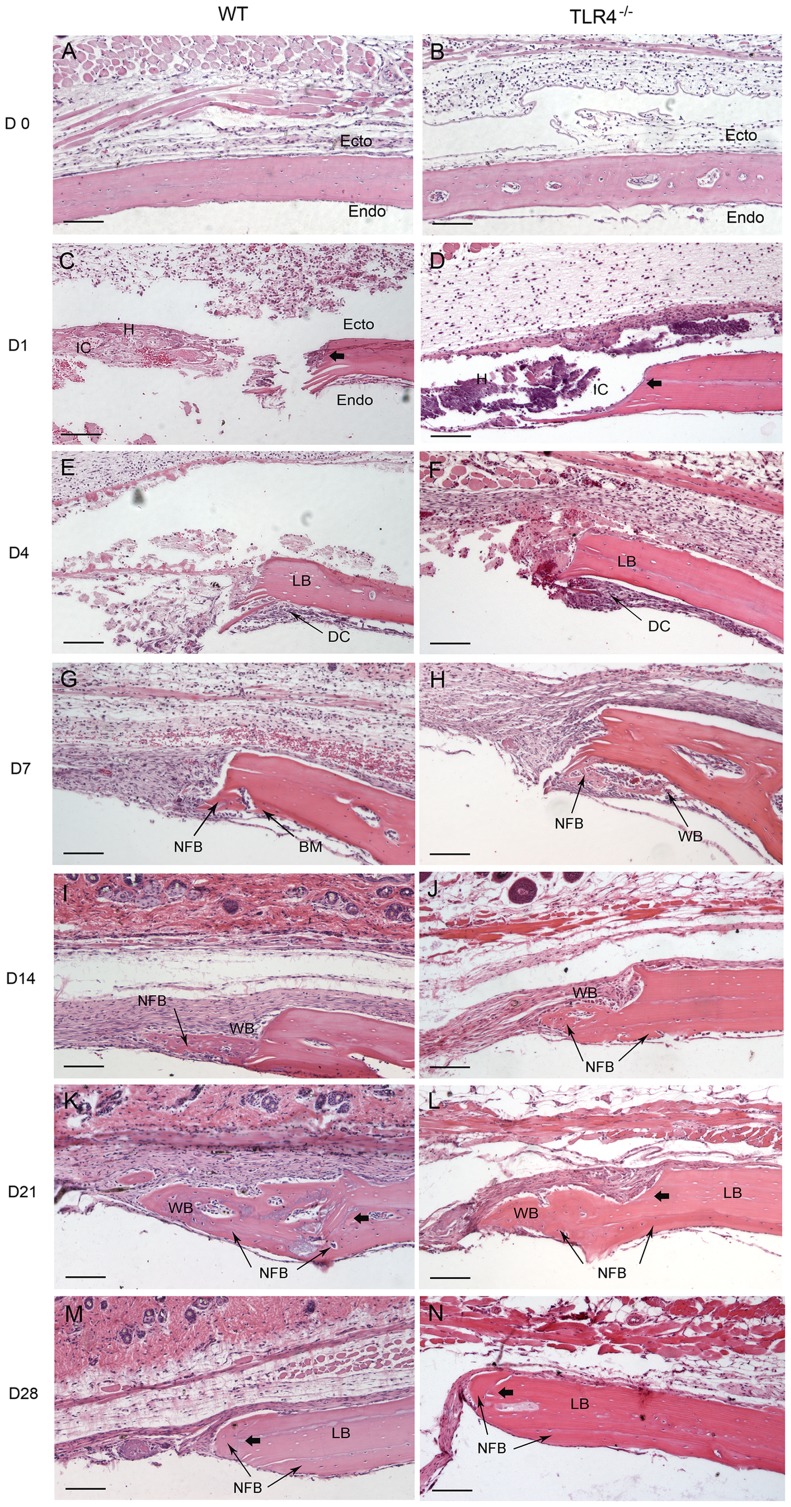
Histophotomicrographs for H&E stained tissues at the defect margins at postoperative time points. WT and TLR4^−/−^ mice showed similar histological staining patterns on days 0, 1 and 4, while larger areas of newly-formed bone were seen in TLR4^−/−^ mice than in WT mice on day 7. Newly-formed cellularized bone matrix was observed on the endocortical (dural) side of the calvarial bone lateral to the defect perimeter in both groups since day 7. Active bone formation was suggested by the presence of large regions of woven bone matrix at the defect margin in both groups on days 14 and 21. Defects in WT and TLR4^−/−^ mice were histologically similar since day 21. (scale bar: 100 µm; bolded arrows: defect margin; endo: endocortical surface of calvarial bone; ecto: ectocortical surface of calvarial bone; H: hematoma; IC: infiltrating cells; LB: lamellar bone; WB: woven bone; NFB: newly-formed bone; DC: dural cells).

**Figure 2:**
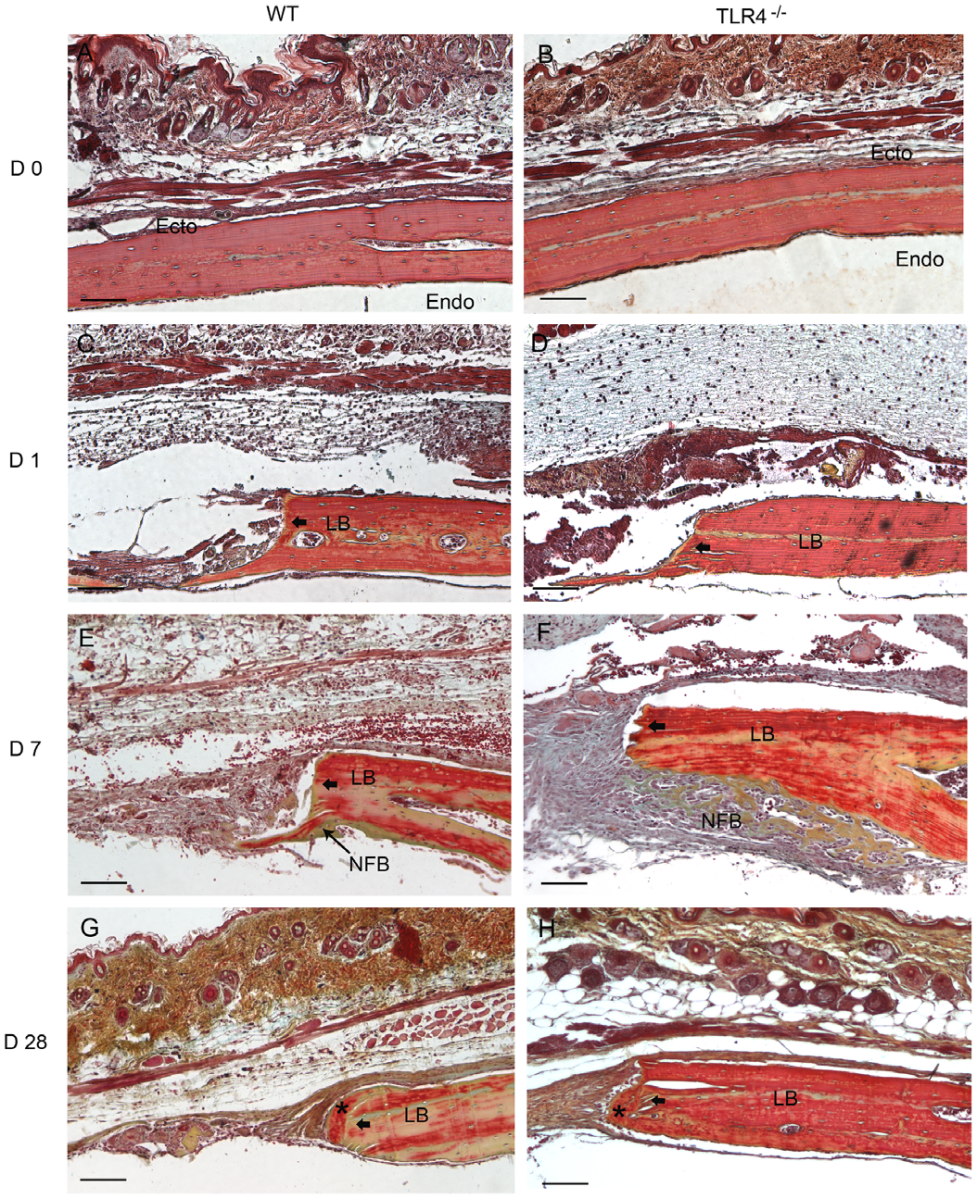
Histophotomicrographs of slides stained with OPN antibodies using immunohistochemistry. OPN staining intensity were more pronounced in TLR4-/- mice than in WT mice on postoperative days 4 and 7. Typical large, rounded osteoblasts were recognized on the surface of the newly-formed bone in both groups on day 14. On day 28, less OPN positive staining was detected in both WT and TLR4^−/−^ mice. (scale bar: 100 µm; bolded black arrows: defect margin; LB: lamellar bone; WB: woven bone; OPN(+): OPN positive stains; OB: osteoblasts).

Day 0: Similar histological staining was shown in WT and TLR4^−/−^ mice on day 0 using H&E and Pentachrome stain (Figure 1A, B; Figure 2A, B).

**Figure 3:**
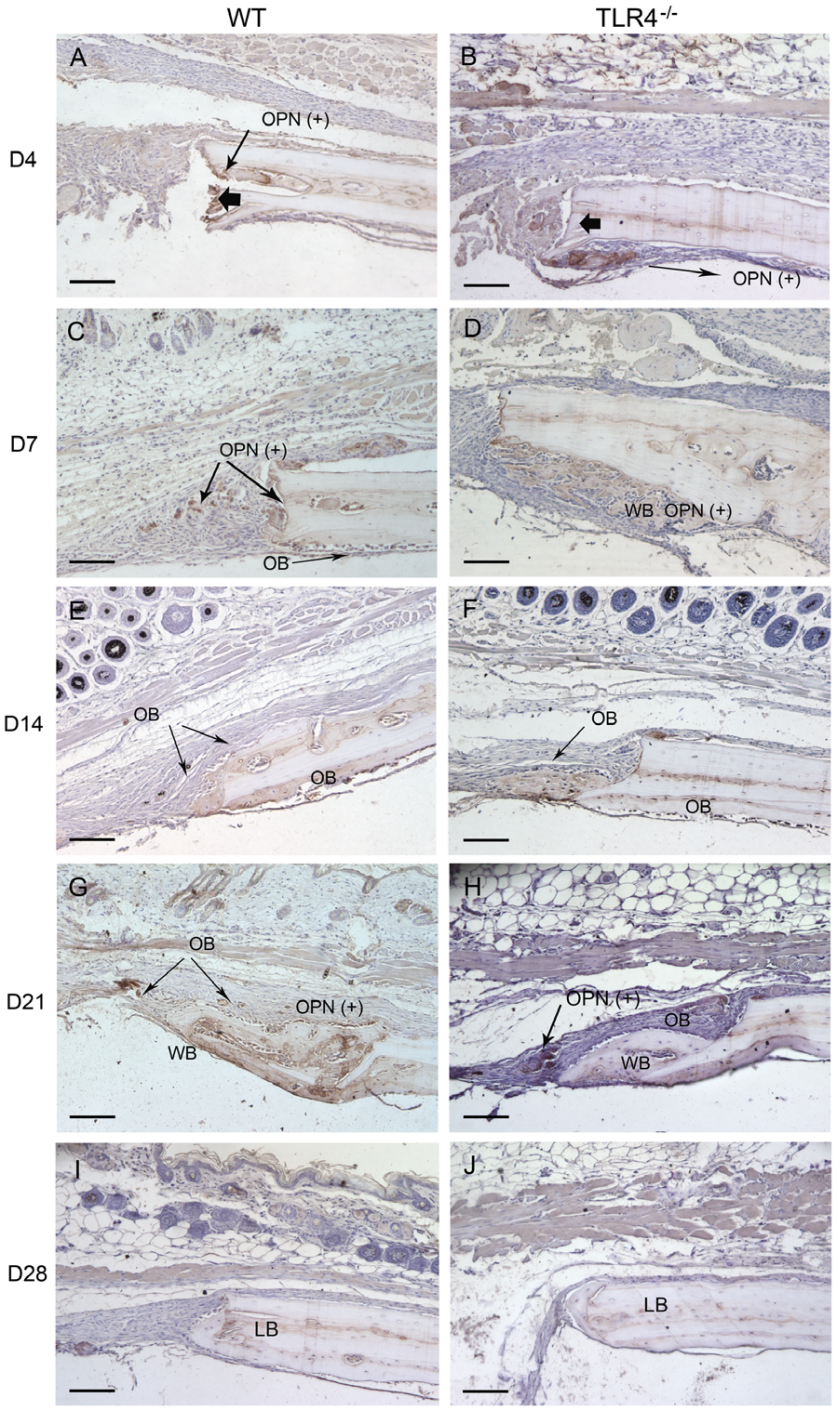
Histophotomicrographs of pentachrome stained tissues in WT and TLR4^−/−^ mice. Similar stains were observed between WT and TLR4^−/−^ mice on day 0 and day 1. An increased amount of newly-formed bone was observed in TLR4^−/−^ mice at day 7, suggesting accelerated healing compared to WT. Lamellar bone (*), which stains positive for acid fuchsin (red) was observed in both groups on day 28, suggesting maturation and remodeling of the newly formed bone matrix. (scale bar: 100 µm; bolded black arrows: defect margin; LB: lamellar bone; NFB: newly-formed bone).

Day 1-day 4: WT and TLR4^−/−^ mice showed similar histological staining patterns on day 1 and day 4 (Figure 1C, D; Figure 2C, D). Hematoma was visible and infiltration by multiple cell types was observed in H&E stained images. The bone defect was filled with hematoma as early as day 1. No changes were observed in the periosteal regions on day 1 (Figure 1C, D). Evidence of hematoma degradation and a reduction in cellular infiltrate was apparent by day 4. On the endocortical surface (presumably derived from the dura mater), cells gathered along the perimeter of the defect (Figure 1E, F). Dural cell layer was thicker and OPN staining intensity were more pronounced in TLR4^−/−^ mice than in WT mice (Figure 3A, B). No bone formation was evident on day 4.

Day 7-day 14: Larger areas of newly-formed bone were seen in TLR4^−/−^ mice than in WT mice on day 7. Newly-formed cellularized bone matrix, indicated by positive saffron yellow staining (Figure 2E, F), was observed on the endocortical (dural) side of the calvarial bone lateral to the defect perimeter in both WT and TLR4^−/−^ mice. The thickness of the dural cell layer was diminished relative to day 4, and there was evidence of active bone formation near the dural side of the calvaria. Disorganized loose connective tissue completely filled the bone defect on day 7 (Figure 1G, H). OPN immunoreactivity was observed along the ectocortical bone surface (periosteal side), along the endocortical bone surface (dural side), within the intercortical region (bone marrow or diploic space), and along the defect margins. OPN immunostaining was more intense in TLR4^−/−^ mice than WT mice (Figure 3C, D). Typical large, rounded osteoblasts were recognized on the surface of the newly-formed bone in WT and TLR4^−/−^ mice on day 14 (Figure 3E, F). Regenerated bone was seen along the dural surface of the calvarial bone and along the defect perimeter. Periosteal thickness remained unchanged between days 7 and 14, while the loose connective tissue was thinner and more organized (Figure 1I, J).

Day 21-day 28: Defects in WT and TLR4^−/−^ mice were histologically similar during this period. On day 21, active bone formation was suggested by the presence of large regions of woven bone matrix at the defect margin in WT and TLR4^−/−^ mice (Figure 1K, L). Mature OPN-positive osteoblasts were observed on the surfaces of the woven bone. Dural cell layers returned to day1 thickness without OPN positive staining (Figure 3G, H). On day 28, less OPN positive staining and less bone formation was detected in both WT and TLR4^−/−^ mice (Figure 1M, N; Figure 3I, J). Bone remodeling was evident with acid fuchsin red positive staining (Figure 2G, H). Periosteum and soft connective tissue became much thinner, more dense, and better organized (Figure 1M, N).

### Quantitative histologic analysis

**Figure 4:**
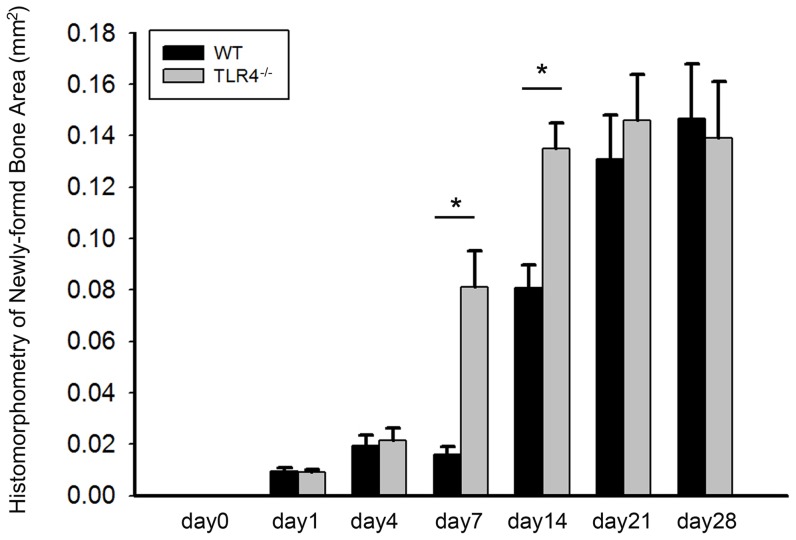
Graph showing newly-formed bone areas measured from H&E stained histology slides. No obvious bone healing was seen before day 7. Significant differences in bone healing areas were observed between WT and TLR4^−/−^ mice on days 7 and 14 postoperatively. Newly-formed bone areas were not significantly different after day 21 in WT mice or after day 14 in TLR4^−/−^ mice (mean +/− SEM ; *p<0.05).

No obvious bone formation was observed in either group before day 7. Two-way ANOVA comparing mean newly-formed bone areas showed a significant group by time point interaction (F = 3.476; *p*<0.05) and significant group (F = 5.946; *p*<0.05) and time point (F = 14.728; *p*<0.05) effects. One-way ANOVA showed no significant differences in bone healing between days 21 and 28 in WT mice, or between days 14, 21, and 28 in TLR4^−/−^ mice (*p*>0.05). Independent T-tests showed significant differences in newly-formed bone areas between WT and TLR4^−/−^ mice on both days 7 and 14 (*p*<0.05, Figure 4). Larger newly-formed bone areas were observed in TLR4^−/−^ mice on days 7 and 14, although comparable levels of bone healing were observed in both groups at other time points (*p*>0.05; Figure 4).

### Radiographic analysis

**Figure 5:**
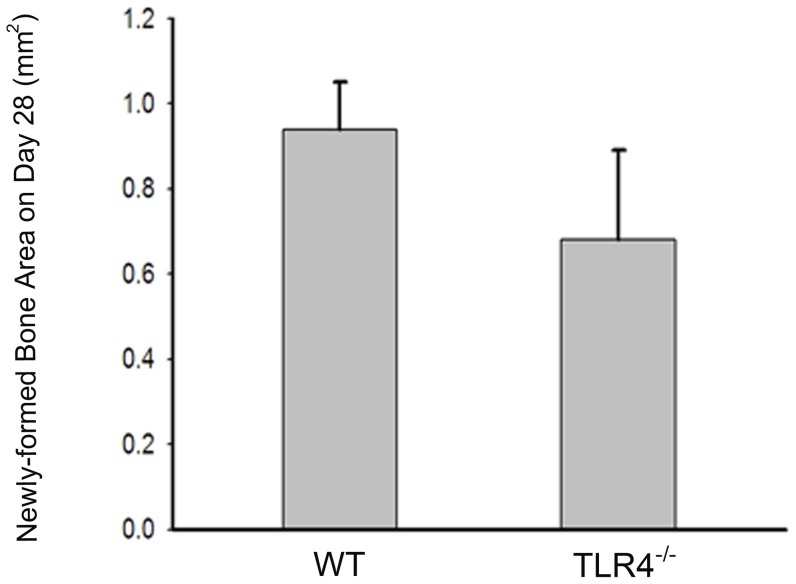
Graph showing similar amounts of radiographically opaque tissue observed on day 28 in both groups. Independent t-test showed no significant differences between the calvarial healing of WT and TLR4^−/−^ mice at this time point. Out of 20 mice tested, none showed complete healing during the 28 days of observation. (mean +/−SEM; n = 10 each).

Mineralized tissue was observed on day 28 radiographs in WT and TLR4^−/−^ mice, although healing remained incomplete during the 28 days of observation. Calculation of newly-formed bone area as determined by radiography revealed healing percentages of 33.14% and 26.83% (data not shown) in WT and TLR4^−/−^ mice, respectively. Independent T-test showed no significant difference in bone healing area between WT (0.9378+/−0.1131 mm^2^; 33.14% healing) and TLR4^−/−^ (0.6814+/−0.209 mm^2^; 26.83% healing) mice on day 28 (*p*>0.05, Figure 5).

### Gene expression

**Figure 6:**
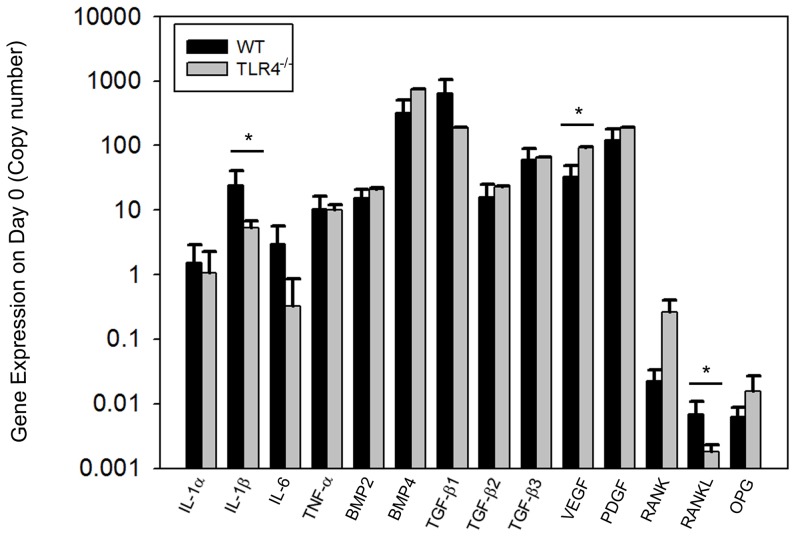
Graph showing quantitative gene expression on day 0 (untreated control) bone tissue. Siginificant differences were identified in the expression of IL-1β,VEGF and RANKL between the two groups. IL-1β and RANKL expression were higher in WT mice and VEGF expression was higher in TLR4^−/−^ mice on day 0 (mean +/−SEM; n = 5 to 7; *p<0.05).

Baseline expression of 14 genes (IL-1α, IL-1β, Il-6, TNF-α, BMP2, BMP4, TGF-β1, TGF-β2, TGF-β3, VEGF, PDGF, RANK, RANKL, and OPG) were measured by quantitative RT-PCR using day 0 untreated samples. Expression of IL-1β and VEGF was significantly different between the two groups.IL-1β (24.52+/−16.79 copy in WT; 5.30+/−1.46 copy in TLR4^−/−^) and RANKL (6.90e-3+/−4.10e-3 copy in WT;1.80e-3+/−5.00e-4 copy in TLR4^−/−^) expression were higher in WT mice, and VEGF (32.42+/−15.90 copy in WT; 92.39+/−1.64 copy in TLR4^−/−^) expression was higher in TLR4^−/−^ mice on day 0 (Figure 6).

**Figure 7:**
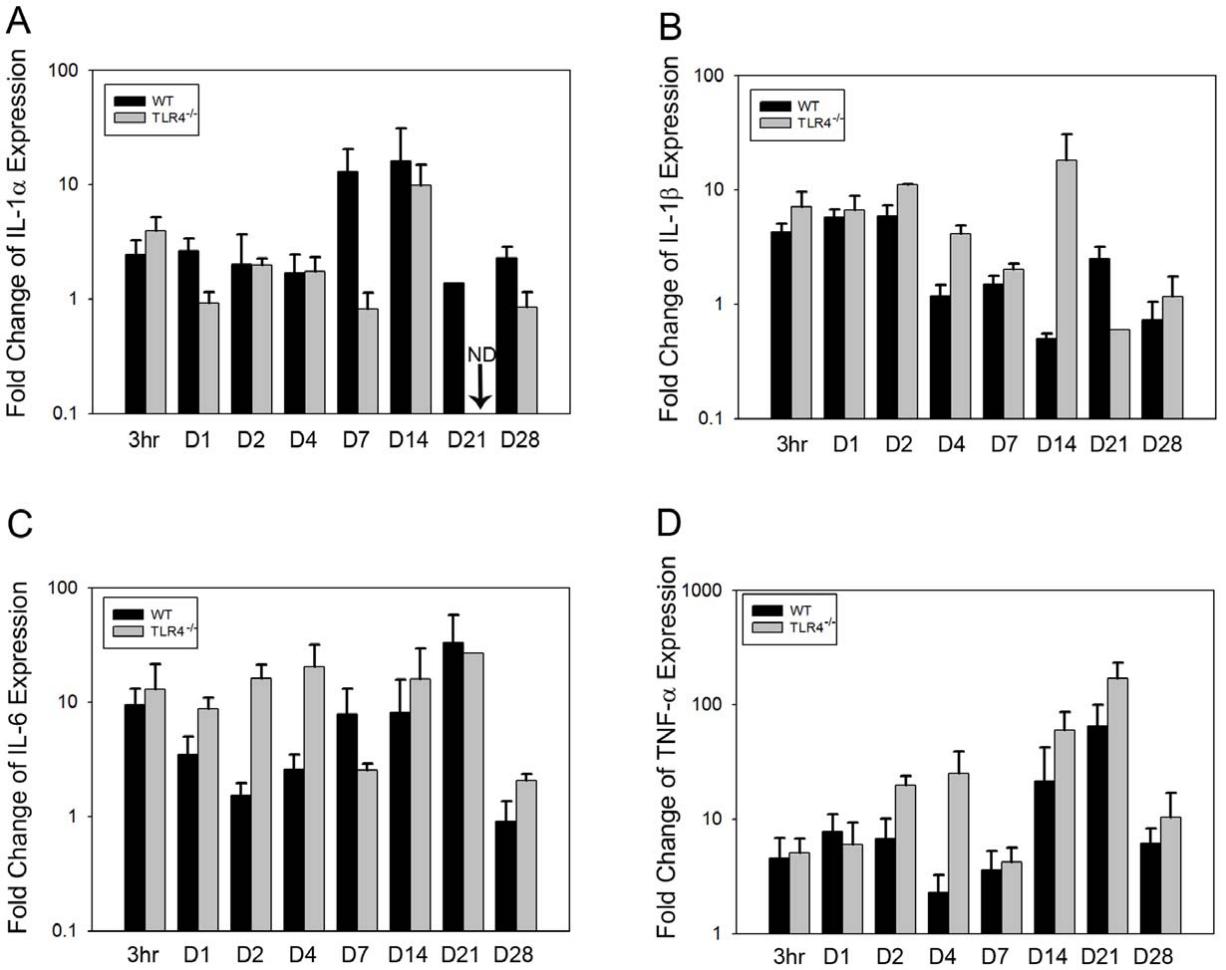
Graph showing relative fold change expression of inflammatory cytokines for WT and TLR4^−/−^ mice (mean fold change over day 0; ND, not detectable). The fold change patterns in IL-1α, IL-1β, and TNF-α expression were similar in both groups. IL-6 had a different expression pattern in TLR4^−/−^ mice compared to WT mice at early time points (mean +/−SEM; n = 5 to 7).

Changes in gene expression at designated time points were determined by relative RT-PCR. The gene expression patterns for IL-1α, IL-1β and TNF-α expression were similar in both groups. Expression of these cytokines increased after surgery, remained stable for a few days and then was upregulated on day 14 and day 21 postoperatively. Significantly higher expression of IL-1β was detected by two-way ANOVA analysis at both early (F = 8.414, p<0.05) and late (F = 26.17, *p*<0.001) time points in TLR4^−/−^ than in WT mice. TNF-α expression was significantly higher at early time points in TLR4^−/−^ mice compared to WT mice (F = 6.451, *p*<0.05) (Figure 7A, B, D). IL-6 exhibited a different expression pattern in TLR4^−/−^ mice, and showed significantly higher fold change expression than in WT mice at early time points postoperatively (F = 6.685, *p*<0.05) (Figure 7C).

**Figure 8:**
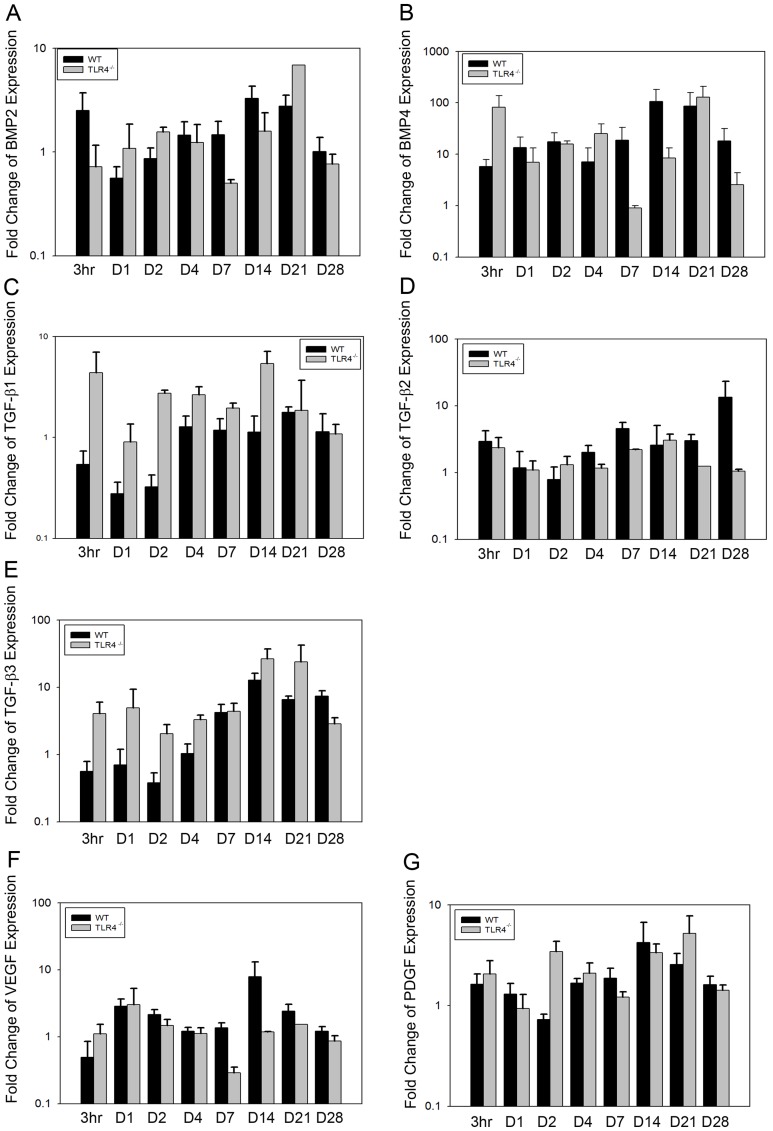
Graph showing relative fold change expression of growth factors for WT and TLR4^−/−^ mice (mean fold change over day 0; ND, not detectable). Similar expression patterns of BMP2, BMP4, TGF-β2, VEGF, and PDGF were observed between the two groups. Higher expression levels of TGF-β1 and TGF-β3 were detected in TLR4^−/−^ mice than in WT mice at early time points (mean +/−SEM; n = 5 to 7).

BMP2 expression increased slightly after surgery in both groups, peaking at day 14 (3.28+/−1.02 fold in WT;1.59+/−0.81 fold in TLR4^−/−^) and declining thereafter. No significant difference in BMP2 expression was observed between the two groups (Figure 8A). BMP4 expression was similar in both groups, increasing slightly until day 21 (87.06+/−72.08 fold in WT; 128.27+/−80.39 fold in TLR4^−/−^) and declining at day 28 (18.24+/−13.53 fold in WT; 2.56+/−1.77 fold in TLR4^−/−^). No significant difference in BMP4 expression was observed between the two groups (Figure 8B).

Significant differences of TGF-β1 were observed in TLR4^−/−^ mice compared to WT mice at early time points (F = 22.636, *p*<0.001) (Figure 8C). No significant differences were detected in the expression of TGF-β2 between groups (Figure 8D). A similar pattern in TGF-β3 expression was observed in both groups. Expression remained stable between 3 h and day 7, and achieved a peak in expression at day 14 (12.69+/−3.55 fold in WT; 26.42+/−10.58 fold in TLR4^−/−^). While the pattern of TGF-β3 expression was similar between the two groups, significantlly higher expression of TGF-β3 was observed in TLR4^−/−^ mice than in WT mice (F = 15.283, *p*<0.001)(Figure 8E). The fold change expression patterns of VEGF and PDGF in WT and TLR4^−/−^ mice were similar. Expression of VEGF in WT and TLR4^−/−^ remained relatively constant until it was upregulated at day 14 (7.90+/−5.25 fold in WT;1.17+/−0.02 fold in TLR4^−/−^), and began to decrease thereafter. Significantly lower expression of VEGF was detected in TLR4^−/−^ mice than in WT mice (F = 31.258, *p*<0.001). PDGF expression was stable in both WT and TLR4^−/−^ mice at all time points. Significantly higher expression of PDGF was observed in TLR4^−/−^ mice compared to WT mice at early time points (F = 4.157, *p*<0.05) (Figure 8F, G).

**Figure 9:**
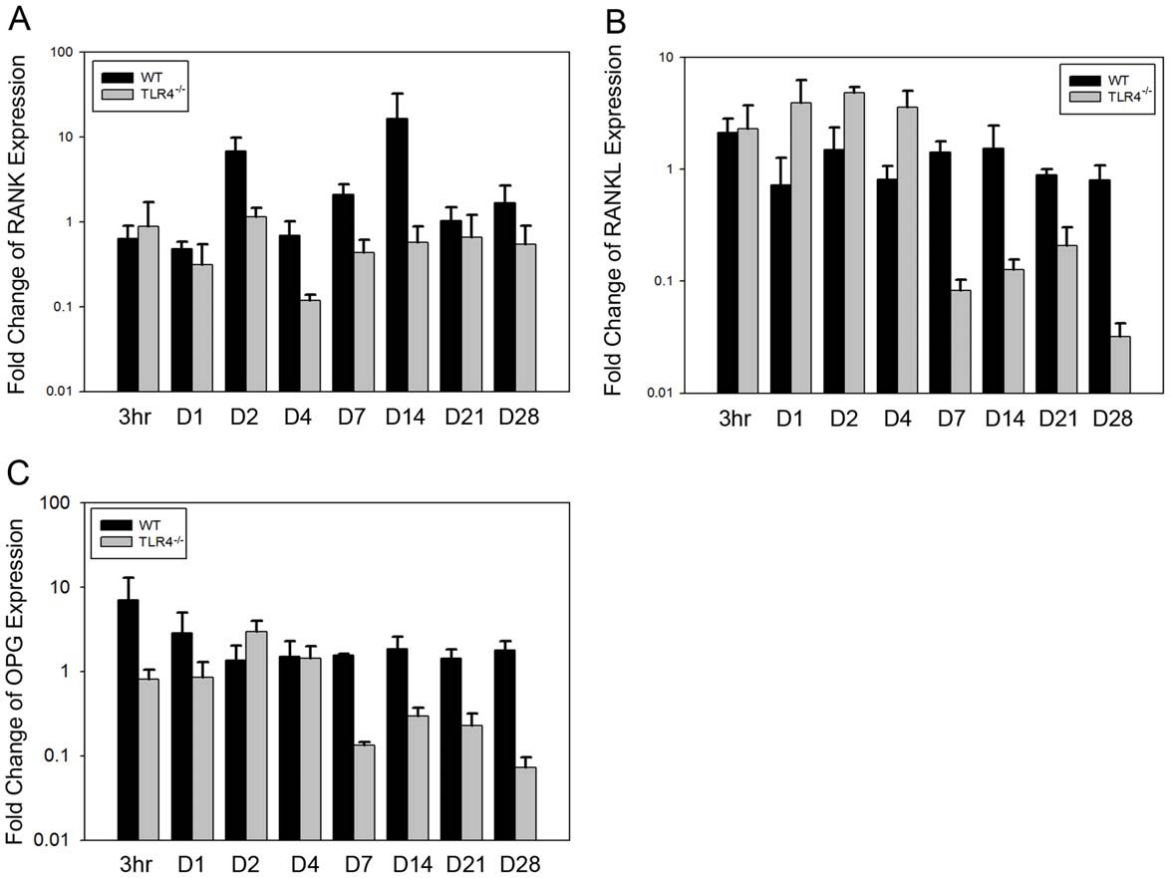
Graph showing relative fold change expression of RANK, RANKL and OPG for WT and TLR4^−/−^ mice (mean fold change over day 0; ND, not detectable). Expression levels of RANK, RANKL and OPG were realtively constant in WT at all time points. Greater variation of the three genes was detected in TLR4^−/−^ (mean +/−SEM; n = 5 to 7).

High expression level of RANK was detected on day 14 in WT mice (16.50+/−15.94 fold), while expression level in TLR4^−/−^ was similar at all time points. Fold change expression of RANK in TLR4^−/−^ was significantly lower than in WT mice at both early (F = 4.453, *p*<0.05) and late time points (F = 6.522, *p*<0.05) (Figure 9A). Expression patterns of RANKL and OPG were relatively stable in WT mice. In TLR4^−/−^ mice, both RANKL and OPG were slightly increased at early time points (≤4 days) and decreased at day 7. Expression level of RANKL was significantly higher in TLR4^−/−^ mice at early time points (F = 4.199, *p*<0.05) and significantly lower at late time points compared to WT mice (F = 56.217, *p*<0.001). Fold change expression of OPG in TLR4^−/−^ was significantly lower than in WT mice at late time points (F = 83.961, p<0.001). (Figure 9B, 9C).

## Discussion

Inflammation has a complex role in fracture repair, although the underlying mechanisms remain unclear [15]–[17]. TLR4 is a member of a highly conserved receptor family and is a critical activator of the innate immune response after tissue injury. TLR4 signaling has been shown to regulate both the immune response and bone metabolism during long-bone fracture healing. Here, we tested the hypothesis that TLR4 activation limits the healing of calvarial defects.

In this study, a smaller than “critical-size” calvarial defect model was used, which by definition, would be expected to heal spontaneously over the duration of the observation. However, none of our 1.8 mm diameter defects healed complete during 28 days of observation. The maximum healing was approximately 30% of the original defect area, which is consistent with previous observations by Gosain et al., who observed 27–35% healing of critical-size defects at 4 weeks [18]. Importantly, we found that no significant healing occurred after 21 days in this model. Pentachrome staining of day 28 defects supports this observation. The newly-formed woven bone adjacent to the defect perimeter stained with acid fuchsin red, suggesting that bone remodeling had already taken place by day 28. OPN reactivity suggests that bone formation, which was highly active around day 21 in WT mice and day 14 in TLR4^−/−^ mice, became less active and less OPN-positive staining was observed. Thus, there may exist a temporal window for mature calvarial healing even in defects smaller than “critical-size”. The molecular signaling involved in the cessation of bone formation within such bone defects need to be further characterized.

Although no significant difference in total calvarial healing was observed between groups radiographically or histologically on post-operative day 28, accelerated healing was observed in TLR4^−/−^ mice. By day 4, a thicker dural cell layer and more dense OPN positive stains were evident in TLR4^−/−^ mice. By day 7, a quantity of newly-formed woven bone was seen on the endocortical calvarial surface in TLR4^−/−^ mice. Quantitative histomorphometry data was consistent with the histological findings mentioned above. Two-way ANOVA comparing mean newly-formed bone areas showed a significant group by time point interaction, suggesting that the WT and TLR4^−/−^ mice healed differently over time. It showed significantly larger area of new bone in TLR4^−/−^ mice on day 7 and day 14. Similarly, improved bone healing has been reported for long bone fracture healing in mice lacking an adaptive immune system. Specifically, in RAG1^−/−^ mice (recombination activating gene 1 knockout), increased bone formation and biomechanical strength at early time points was associated with reduced pro-inflammatory cytokine expression and increased expression of the anti-inflammatory cytokine, IL-10 [7]. Those observations suggest that the suppression of the adaptive immune system might maximize the regenerative and minimize the destructive effects of inflammation which may promote fracture repair [7]. In our model, bone healing was obviously accelerated in TLR4^−/−^ mice, although the total bone healing achieved was comparable between two groups. Whether accelerated calvarial healing effect is due to the suppression of TLR4-mediated inflammatory response requires further exploration.

Gene expression analysis showed that expression of inflammatory cytokines, generally, was elevated 3 hour post-operatively, and a second spike in inflammatory gene expression was detected around day 14 in both groups. Greater variation was detected in the expression of growth factors after surgery. These findings are consistent with the gene expression patterns described in long bone fracture models [19]–[20]. Of greater interest, we found that IL-1β, IL6 and TNF-α,TGF-β1, TGF-β3 and PDGF were more highly expressed at earlier time points in TLR4^−/−^ mice. We speculated that accelerated bone healing observed in TLR4^−/−^ mice might, at least in part, be due to earlier and higher expression of these genes [21]–[23].

The inflammatory cytokines IL-1β, IL-6 and TNF-α have complex effects on bone regeneration. IL-1β can stimulate osteoblasts and bone matrix formation, but also suppress the differentiation of mMSCs [24]. In vivo studies in IL1α, IL1β, and IL1α/β mutant mice have shown impaired osteoclast development to result in increased bone density and femoral bone mass within these animal models [25]. IL-6 has been shown to both promote and inhibit osteoclastogenesis [26]–[27]. An in vivo study determined that the absense of IL-6 promoted inflammatory calvarial bone loss in a mouse model [28]. Although IL-6^−/−^ mice showed reduced osteoclastogenesis and impaired callus strength at the early time points after surgery, similar long bone healing outcomes were observed in comparison with WT mice after 3 weeks [29]. Therefore, the role of IL-6 in osteoclastogenesis has been more elucidated [28]–[29]. Further studies are needed to understand the impact of TLR4-mediated inflammatory and related inflammatory cytokines expression on calvarial bone repair.

RANK, OPG and RANKL are important mediators of remodeling during bone formation and bone regeneration. TLR signaling inhibits RANKL-mediated osteoclastogenesis, although TLR activation can also promote osteoclastogenesis by inducing RANK and TNF-α expression in osteoblasts [9]. In the present study, significant differences were apparent in the expression of RANK, OPG and RANKL between TLR4^−/−^ and WT mice. Expression of RANKL was significantly higher in TLR4^−/−^ mice compared to WT mice at early time points. RANK, RANKL and OPG all showed significantly lower expression in TLR4^−/−^ mice than in WT mice at late time points. During long bone regeneration, upregulated RANKL at primary long bone formation stage is associated with mineralized cartilage resorption and bone formation, and downregulated expression indicated bone remodeling. Early high expression levels in TLR4-/- mice suggest early and high resorption activity of osteoclasts. The differential expression of these three genes might explain the observed accelerated bone formation and early bone remodeling in TLR4^−/−^ mice. It remains unclear whether differenes in RANK, RANKL and OPG expression are directly linked to the absence of TLR4 signaling.

Conclusion: The present study revealed accelerated bone formation and bone remodeling in absence of TLR4 signaling pathway. This phenotype is also associated with changes of local inflammatory cytokines and osteoclastogenic factors expression. Further work is required to determine whether regenerative effects of inflammation mediated by absence of TLR4 may lead to accelerated skull bone repair.
